# Aortic Valve Engineering Advancements: Precision Tuning with Laser Sintering Additive Manufacturing of TPU/TPE Submillimeter Membranes

**DOI:** 10.3390/polym16070900

**Published:** 2024-03-25

**Authors:** Vlad Ciobotaru, Marcos Batistella, Emily De Oliveira Emmer, Louis Clari, Arthur Masson, Benoit Decante, Emmanuel Le Bret, José-Marie Lopez-Cuesta, Sebastien Hascoet

**Affiliations:** 1 Centre Hospitalier Universitaire de Nîmes, Service de Radiologie, Imagerie Cardiovasculaire, 4 Rue du Professeur Robert Debré, 30900 Nîmes, France; 2 Hôpital Marie Lannelongue, Groupe Hospitalier Paris Saint Joseph, Faculté de Médecine Paris-Saclay, Université Paris-Saclay, Inserm UMR-S 999, BME Lab, 133 Avenue de la Résistance, 92350 Le Plessis Robinson, France; b.decante@ghpsj.fr (B.D.); e.lebret@hml.fr (E.L.B.); s.hascoet@ghpsj.fr (S.H.); 3 3DHeartModeling, 30132 Caissargues, France; 4 Polymers Composites and Hybrids Department, IMT Mines Alès, 30319 Ales, France; marcos.batistella@mines-ales.fr (M.B.); emily.de-oliveira-emmer@mines-ales.org (E.D.O.E.); louis.clari@mines-ales.org (L.C.); arthur.masson@mines-ales.org (A.M.); jose-marie.lopez-cuesta@mines-ales.fr (J.-M.L.-C.)

**Keywords:** thermoplastic polyurethane (TPU), thermoplastic elastomer (TPE), shape memory polymer, aortic valve, valve substitute, surgical training, TEHV

## Abstract

Synthetic biomaterials play a crucial role in developing tissue-engineered heart valves (TEHVs) due to their versatile mechanical properties. Achieving the right balance between mechanical strength and manufacturability is essential. Thermoplastic polyurethanes (TPUs) and elastomers (TPEs) garner significant attention for TEHV applications due to their notable stability, fatigue resistance, and customizable properties such as shear strength and elasticity. This study explores the additive manufacturing technique of selective laser sintering (SLS) for TPUs and TPEs to optimize process parameters to balance flexibility and strength, mimicking aortic valve tissue properties. Additionally, it aims to assess the feasibility of printing aortic valve models with submillimeter membranes. The results demonstrate that the SLS-TPU/TPE technique can produce micrometric valve structures with soft shape memory properties, resembling aortic tissue in strength, flexibility, and fineness. These models show promise for surgical training and manipulation, display intriguing echogenicity properties, and can potentially be personalized to shape biocompatible valve substitutes.

## 1. Introduction

The continuous demand for advanced materials in biomedical applications has led to significant expansion in their development [1]. One area that has garnered a lot of research attention is tissue-engineered heart valves (TEHVs), aiming to create structures that combine biocompatibility, hemocompatibility, non-immunogenicity, bioactivity, accurate reproduction of architecture, and replication of essential physical characteristics [2].

In this setting, current research trends focus on developing and characterizing bioactive materials designed not only to be biocompatible and non-immunogenic but also to promote extracellular matrix production and interact with the implantation environment, producing a beneficial response regarding local inflammation. Indeed, while natural polymers like collagen and fibrin offer excellent biocompatibility and bioactivity [3], they may lack sufficient mechanical strength. Hence, there is growing interest in synthetic biomaterials for tissue-engineered heart valve (TEHV) manufacturing [4].

Current research focuses on exploring various adaptable synthetic materials, their composition, and methods for adjusting their mechanical properties.

Additionally, it delves into technologies for producing valvular scaffolds.

Poly(glycerol sebacate) (PGS) is an example of a synthetic polymer utilized in this field. A biocompatible and biodegradable material, copolymers of poly(glycerol sebacate) and poly(caprolactone) resilient to cyclic mechanical stress were utilized in creating valvular scaffolds via melt molding [5].

Other elastomers like thermoplastic polyurethanes (TPUs) and thermoplastic elastomers (TPEs) have been applied to tissue-engineered heart valve (TEHV) applications [6].

TPEs, particularly those based on the structure of styrene-isobutylene-styrene (SIBS), are biocompatible copolymers [7] that demonstrate high flex fatigue and stability, making them suitable for producing synthetic heart valves [8].

On the other hand, polyurethanes (TPUs) offer high tunability concerning structure and mechanical properties. They exhibit characteristics such as high shear strength, elasticity, durability, fatigue resistance, stability with high memory shape, and improved handling characteristics [9].

Applications of polyurethanes in biomedicine are varied. They include applications such as antibacterial surfaces, blood oxygenators, dialysis devices, vascular stents, vascular prostheses, and diverse tissue engineering scaffolds for either hard or soft tissue: bone, cartilage, skeletal muscle, heart, or vessels [10].

TPUs serve well as electrical insulators in durable cardiac pacing leads [11]. Additionally, the incorporation of fillers such as carbon black or silica further enhances the insulating properties of the composite [12]. Conversely, the addition of aluminum silicate improves the dielectric properties.

Optimal for sterilization, they exhibit high biocompatibility and are non-thrombogenic [13]. This allows for unrestricted use in blood-contacting devices, from central venous catheters to heart assist valves [6].

New developments in polyurethane structure have involved shifting from aromatic to aliphatic compositions [14] to enhance biocompatibility and have explored formulations like polyester urethanes, polyether urethanes, polycarbonate urethanes, and polyether urethane urea in heart valve fabrication. Polycarbonate urethanes are emerging for their lower rates of calcification and biodegradation, enabling thinner leaflet fabrication. Techniques like dip coating and drop coating generate multilayer configurations with differing stiffnesses [15].

Polyurethane elastomers, with dynamic dimethylglyoxime-urethane groups, promote mutual healing between broken parts by reformulating bonds, representing promising developments with intriguing applications like aortic aneurysm wrapping in murine models [16].

Pure polyurethanes (TPUs) exhibit high resistance against hydrolytic degradation. However, incorporating degradable links increases their susceptibility to hydrolysis [17].

The mechanical properties of polyurethanes are closely linked to their composition, which consists of hard and soft segments. The hard segment is formed by diisocyanate and a chain extender, while the soft segment typically comprises polyester, polyether, or polycarbonate diol [6].

Adjusting mechanical properties is a crucial research focus in tissue-engineered heart valves (TEHVs) to align with the aortic valve’s strength, elasticity, and mimicking leaflet anisotropy (radial/circumferential).

To address the relatively high stiffness of TPUs [18], modifications have been made to polyurethane structures by adjusting the soft/hard segment ratio or by incorporating poly(ester urethane) urea (PEUU) [19] or mixed TPU–silicone copolymers [20].

Another method of adjusting mechanical properties is through the manufacturing process itself.

The aim of our study was precisely to adjust the mechanical characteristics of thermoplastic polyurethanes (TPUs) and thermoplastic elastomers (TPEs) using a method called selective laser sintering (SLS). Through this technique, we were able to modify exposure parameters to tune these properties thoroughly [21].

The second goal of our study was to produce micrometer-thick membranes designed specifically for aortic valve scaffolds. This is a crucial aspect of tissue-engineered heart valves, which is inherently difficult [22]. Submillimeter leaflets in a polymeric valve can significantly reduce energy loss within the valve, potentially enhancing durability and minimizing destructive energy effects [23].

Various methods have been employed for this purpose, including melt molding [24], electrospinning, and electro-spraying [25], as well as stereolithography or 3D printing of photo-curable silicones by direct ink writing [26].

We propose in this study that selective laser sintering (SLS) [27] holds promise in this context as well, offering customizable and quickly tunable valvular scaffolds with high spatial resolution [28], and could be suitable for creating submillimeter membranes.

We aimed to explore SLS process parameters for both TPUs and TPEs, seeking an optimal balance between flexibility and strength to simulate aortic valve tissue and to evaluate the capability of printing aortic valve models with submillimeter membranes.

## 2. Materials and Methods

### 2.1. Thermoplastic Polyurethanes

TPU 1301 powder from EOS (Electro Optical System—EOS^®^ GmbH, Munich, Germany) was used. The particle size distribution parameters were 22 μm for D10, 72 μm for D50, and 138 μm for D90.

The thermal properties of the TPU used and the melting and crystallization temperatures, are shown in Table 1.

The cooling chamber temperature was set to 80 °C, lower than the recrystallization range, while the powder bed temperature was set to 110 °C, just below its melting temperature.

**Figure 1:**
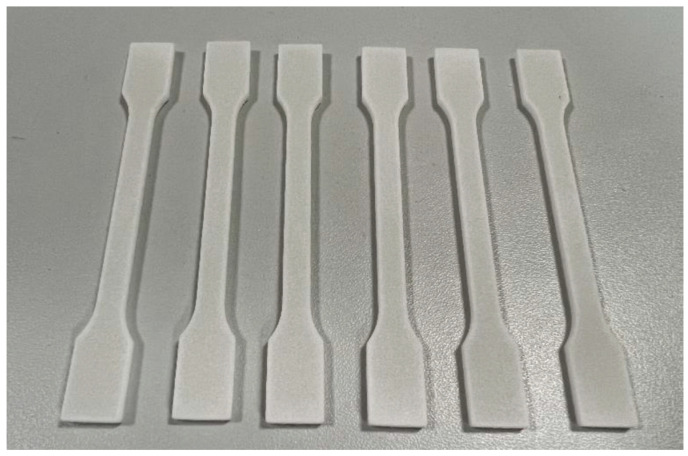
Standard samples with a 2 mm thickness.

ISO 527 1BA standard samples with thicknesses of 2 mm (Figure 1) were fabricated using a FORMIGA P110 (Electro Optical Systems France SAS, Lyon, France). The influence of sample orientation, flat, on edge, 45°, and upright, and exposure parameters on the mechanical properties was assessed. Table 2 outlines the parameters under investigation.

Additionally, 0.5 mm membranes were manufactured and analyzed for mechanical properties.

Several laser power levels were chosen (16/19/22/25 W) with hatching distances of 0.2 mm and 0.25 mm and scan speeds of 2000/2500/3000 mm/s across 19 different samples. The layer thickness was maintained at a constant 0.1 mm. The energy density levels ranged from 0.16 J/mm^3^ to 0.50 J/mm^3^.

**Figure 2:**
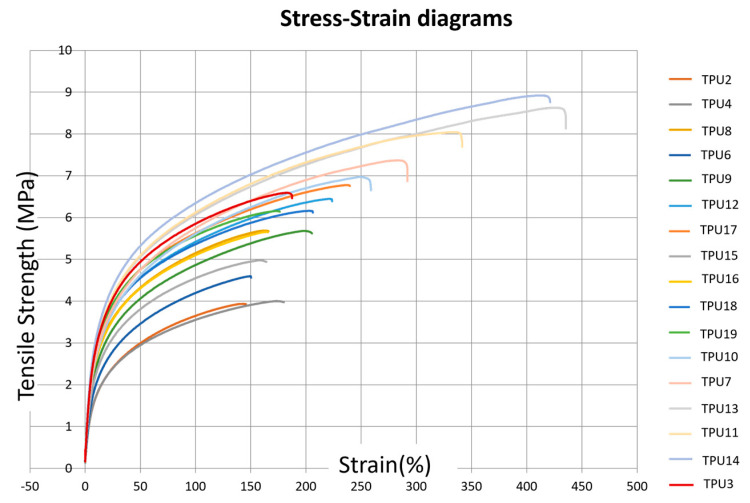
Stress–strain diagrams corresponding to the different TPU presets defined in Table 2.

To assess the feasibility of printing samples with thin walls, which are necessary for valvular models, we printed tensile specimens with a thickness of 0.5 mm. Four laser scan strategies were employed for this purpose. The strategies included low-energy simple contour at 14 W and 16 W. Additionally, two other scanning strategies were incorporated into the experimentation: ‘edge,’ which allows for an extra laser scan at the center of the layer (as shown in Figure 2), and ‘double contour,’ involving two internal contours, utilizing a laser power of 12 W and a speed of 2300 mm/s. This strategy allows us to enhance energy density in thin samples despite a low laser power.

### 2.2. Thermoplastic Elastomers

The elastomer used in this study was TPE 210-S (ALM^®^ Nibionno, Temple, TX, USA), processed at room temperature to prevent powder heterogeneity and the formation of aggregates between powder particles. Samples were manufactured using a Sharebot SnowWhite2 (Sharebot^®^, Nibionno, LC, Italy) 3D printer, with a laser power of 14 watts and a percentage adjustment ranging between 50% and 100% of the total power. The layer thickness was set at 0.12 millimeters, scan line spacing at 0.1 millimeters, and laser speed varied between 50,000 and 10,000 dots per second (dps), where one dot corresponds to 60 microns.

Specimens were manufactured in flat and on-edge positions to apply the maximum energy density (Ed) without compromising the elastomer. However, flames were generated during printing at 100% laser power and a laser speed of 10,000 dots per second (dps), especially for samples positioned flat. Consequently, the on-edge position was favored, resulting in a smaller printed surface area and lower energy density than the flat position with the same parameters (see Supplementary Materials).

Our experiments noted that the mechanical properties of reused powder decreased by almost 50%, prompting the use of only new powder for the models.

### 2.3. Aortic Valve Models

#### CAO File and Aortic Valvular Models

The procedure for obtaining the valve models has been previously described [29]. In brief, we performed intensity-based segmentation using Philips IntelliSpace Portal V11 (Philips, Best, The Netherlands) on cardiac ECG-synchronized CT scan. Subsequently, we obtained a volume rendering of the aortic valve and the ascending aortic root and transformed it into a stereolithography (STL) file. Post-processing of the STL file was conducted offline, utilizing software like Materialize Magics^®^ and Geomagic^®^ 10.0 version.

The model created was a bicuspid aortic valve designed for planning surgical procedures for the patient. Optimized process parameters were employed to fabricate the model, featuring thin walls ranging from 1.3 mm to 0.3 mm. In addition to assessing their mechanical properties, we evaluated their ultrasonic characteristics by immersing the models and conducting ultrasound scans using 3D probes (Philips CVx 3D probe, Best, The Netherlands).

Reproducibility was verified by performing 3D comparisons and scanning prints obtained from replicas using identical presets, explicitly focusing on the intricate details of the aortic leaflets. The average distance measured was 0.04 mm ± 0.3 mm (refer to the supplementary data for the figure), suggesting a highly reproducible process.

Shape memory was assessed by conducting a comparison study of the models after stretching. We performed a scan of the surface of the piece immediately after 50 cycles of full deformation and compared it to the initial STL file of the model.

### 2.4. Tensile Tests

We conducted uniaxial tensile testing on standard 2.0 mm thick specimens using a Zwick Z010 Universal Mechanical Testing Machine (ZwickRoell^®^, Ars-Laquenexy, France) at room temperature. The testing employed a 55 mm gauge length, a 100 mm/min rate for elongation at break, and 1 mm/min with an extensometer to determine the modulus of elasticity.

For the additional 0.5 mm thick TPU specimens, testing was carried out using the same equipment but at a tensile rate of 10 mm/min to determine the strain at break. The grips were modified to better accommodate the thickness of the specimens, which are thin films. Five specimens were tested, and mean values are provided.

### 2.5. Scanning Electron Microscopy (SEM)

The microstructure of the samples was analyzed using an environmental scanning electron microscope (Quanta 200 FEG FEI, Hillsboro, OR, USA). The specimens were subjected to cryogenic (low temperature) fracture by immersing them in liquid nitrogen. Before observation, a thin layer of carbon was sputtered onto the specimens to prevent any electrical charge during the examination.

## 3. Results

### 3.1. TPU Mechanical Properties

#### 3.1.1. Influence of Laser Exposure Parameters on TPU Mechanical Properties

Uniaxial tensile testing on standard 2.0 mm thick specimens for elongation at break and 1 mm/min with an extensometer to determine the modulus of elasticity are shown in Figure 2.

In the SLS technique, key exposure parameters governing energy density (Ed) (J/mm^3^) are defined by Equation (1) [30]:

Ed = (LP × Hs × Ss)/d(1)

where:

1. LP represents the laser power in W;
2. d is the layer thickness (0.1 mm);
3. Ss denotes the laser scan speed in mm/s;
4. Hs signifies the distance between two scan lines in mm.

**Figure 3:**
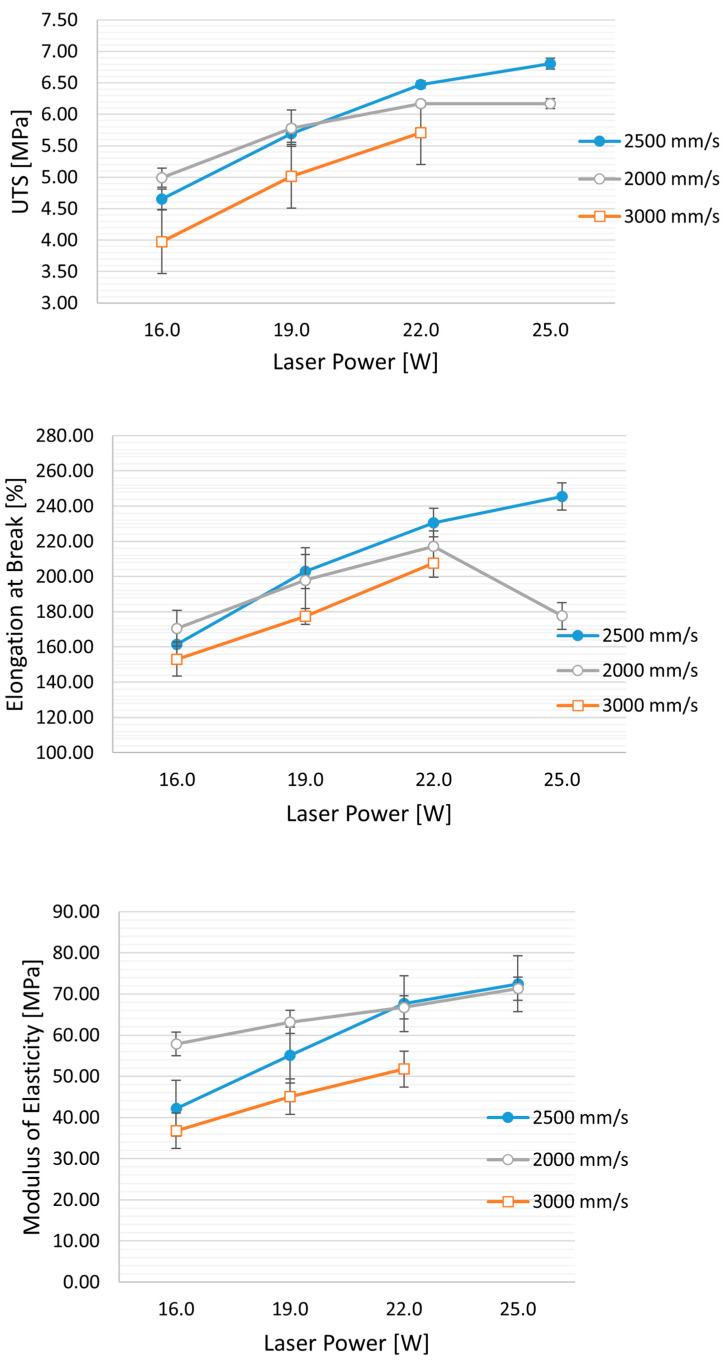
Effect of laser speed and power on mechanical properties: UTS (ultimate tensile strength), elongation at break, and modulus of elasticity. Hs (Hatching distance) is held constant at 0.25 mm, while the laser power varies from 16 W to 25 W and the speed from 3000 mm/s to 2000 mm/s.

The influence of laser power and laser scan speed on the ultimate tensile strength (UTS) and elastic modulus (E) is depicted in Table 3. An increase in UTS and elastic modulus is observed with a decrease in scanning speed (from 3000 mm/s to 2000 mm/s) and with an increase in laser power (from 12 W to 25 W) (Figure 3). This phenomenon is associated with improved powder coalescence, reducing part porosity.

Furthermore, for high values of laser power and lower scan speed (corresponding to an energy density (ED) of 0.50 J/mm^3^), a decrease in mechanical properties, particularly in elongation at break, is evident (see figure). This decrease can be attributed to an excessively high energy density, which may cause the thermal degradation of TPU, leading to the release of volatile polymer fragments and an increase in part porosity. The minimum energy value applied was 0.16 J/mm^3^, resulting from the 12 W laser at 3000 mm/s and an Hs of 0.25 mm. The obtained UTS is 3.28 ± 0.14 MPa, and E is 28.23 ± 0.8 MPa.

Hatching distance (Hs) also significantly impacts tensile strength, nearly doubling as Hs decreases from 0.25 to 0.20 mm. Specifically, the ultimate tensile strength (UTS) increases from 3.79 MPa to 7.2 MPa. The elongation at break improves from 150% to 275%, all while using the same laser power (LP = 16 W) and speed (3000 mm/s), resulting in energy densities (ED) of 0.27 J/mm^3^ and 0.21 J/mm^3^, respectively.

Similarly, there is an increase in elastic modulus (from 36.8 to 63.45 MPa) and elongation at break (from 153% to 272%). Interestingly, when comparing the mechanical properties of samples with similar energy density (ED = 0.26 J/mm^3^: TPU-5, and ED = 0.27 J/mm^3^: TPU-6), reducing Hs results in an approximately 75% increase in UTS (from 4.7 MPa to 7.1 MPa).

**Figure 4:**
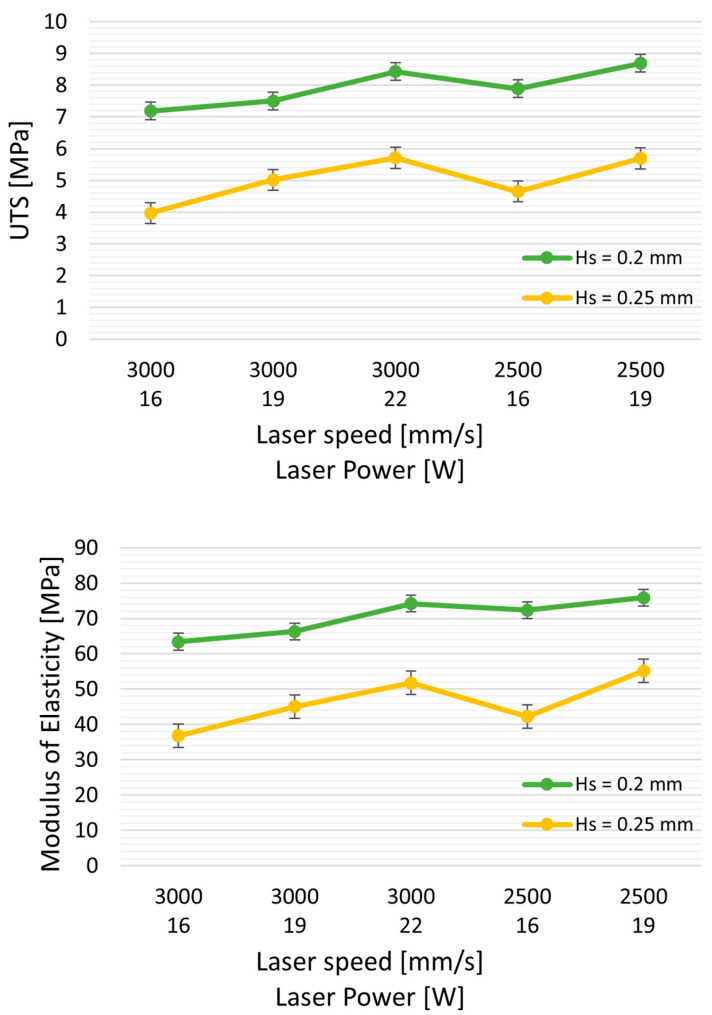

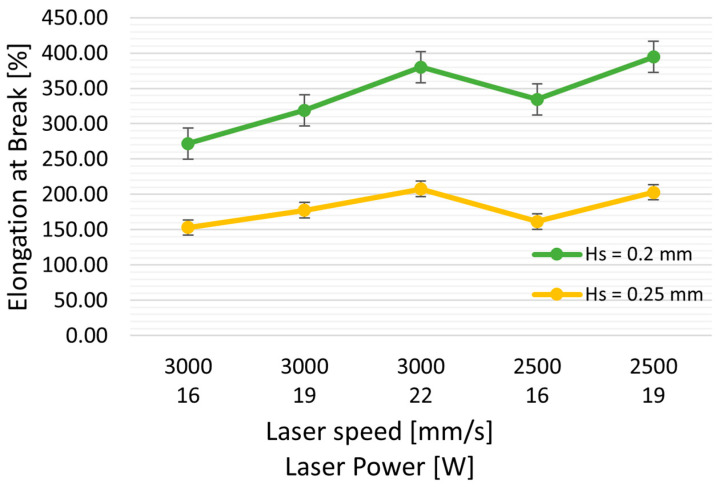
Effect of Hs (hatching distance) on mechanical properties: UTS (ultimate tensile strength), elongation at break, and modulus of elasticity. Green curves represent the properties with Hs = 0.20 mm, and yellow curves represent Hs = 0.25 mm, with varying laser powers and at two speeds, 3000 and 2500 mm/s.

Furthermore, reducing Hs from 0.25 to 0.2 mm appears to have a more pronounced effect than reducing laser power from 22 W to 16 W (as illustrated in Figure 4). Despite a slight decrease in E (TPU-7 with ED of 0.29 J/mm^3^ vs. TPU-6 at 0.27 J/mm^3^), the UTS increased from 5.9 MPa to 7.19 MPa. The reduction in Hs leads to increased spot laser superposition, potentially elevating powder temperature, resulting in improved powder coalescence and reduced part porosity.

#### 3.1.2. Mechanical Characteristics of 0.5 mm TPU Thin Membranes

Table 4 displays the mechanical properties of 0.5 mm thin membranes obtained under various exposure parameters and spatial orientations. The peculiarity of thin specimens is the absence of infill, thus eliminating any effect of the Hs parameter on the specimens due to the laser spot size, which is 0.4 mm for the Formiga P110 equipment.

Compared to the 2 mm specimens, the 0.5 mm membranes are reduced by about 50% in Young’s modulus and 25% in ultimate tensile strength (UTS) when a simple contour is applied (Table 4). However, when employing double contour and edge scan strategies, a local increase in energy density results in higher powder temperatures and better powder coalescence. Consequently, there is a substantial increase in mechanical properties (the E modulus doubles), allowing for a reduction in printed thickness by up to 0.3 mm.

The part’s orientation also significantly affects mechanical properties (Table 4). This behavior can be attributed to weaker cohesion between the superposed layers compared to the X and Y directions, with the most favorable position being on edge. In contrast, the flat position leads to more print failures due to tearing as the roller passes over.

### 3.2. TPE Mechanical Properties

To achieve the study’s objective of obtaining resilient membranes with an E modulus ranging from 2 to 10 MPa, the challenge for TPEs is the opposite of that for TPUs. It involves finding the highest energy to ensure optimal TPE performance without degrading the material.

Reducing the laser speed and increasing laser power, and thus the energy density (Ed), leads to an improvement in the mechanical properties of the TPE (see Table 5). However, if the laser speed decreases too much, it can result in material degradation, as observed in SEM images showing a hollow appearance in the middle of the piece (refer to SEM figure for TPE-6). Further increasing Ed, with 100% laser power and a laser speed of 10,000 DPS (points per second, and a point corresponding to 60 microns), led to the appearance of flames during the printing process.

Conversely, reducing the Ed by increasing speed increases the porosity of the piece, with a substantial negative impact on the mechanical properties (TPE-2). An optimal average energy density was defined (0.64 J/mm^3^) to ensure the best overall properties (modulus E around 2 MPa) without degrading the printed parts (exposure settings: laser power = 100%; % edge 95%, and laser scan speed (Ss) = 30,000 dps).

### 3.3. Scanning Electron Microscopy Analysis

**Figure 5:**
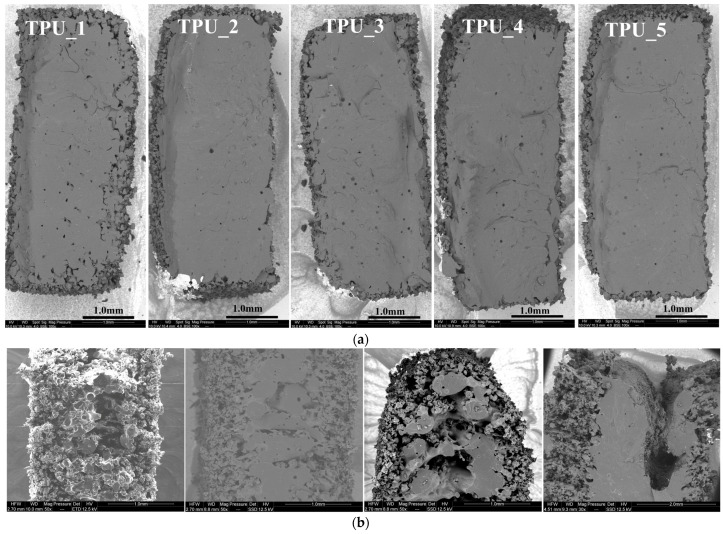
Scanning electron microscopy. (**a**) SEM of TPU as a function of progressively delivered energy density (ranging from TPU_1: 0.21 J/mm^3^, TPU_2: 0.25, TPU_3: 0.26, TPU_4: 0.29, TPU_5: 0.30 J/mm^3^); (**b**) SEM of TPE samples obtained with different levels of energy: Left: Low energy (0.34 J/mm^3^) showing increased rugosity (TPE-2). Second from the left: Optimal energy delivery (0.64 J/mm^3^) for TPE-7. Second from the right and right: Highest energy (0.78 J/mm^3^ and 1.36 J/mm^3^) producing a hole for TPE-6 and TPE-1.

In Figure 5, SEM cross-sectional images depict the microstructure of samples with varying energy densities for both TPUs and TPEs. At a low energy density of 0.21 J/mm^3^, TPU_1 exhibits higher rugosity (Figure 5a), and similar observations are made for TPE at Ed < 0.34 J/mm^3^ (Figure 5b, left). Upon increasing Ed to > 0.65 J/mm^3^, the structure becomes denser. The ‘optimal’ TPE sample (Figure 5b, second from the left) displays a dense center with some peripheral rugosity. However, when Ed exceeds 0.78 J/mm^3^, the center of the piece degrades due to a rise in local temperature (Figure 5b, right).

### 3.4. Application to the Fabrication of Heart Valve Structures

#### 3.4.1. Structural Considerations

Based on previous research, we selected three criteria for modeling cardiac valve structures:

1. Leaflet thickness less than 1 mm;
2. Resistance to repeated bending and shearing;
3. Shape memory: Spontaneous maintenance and recovery of anatomical conformation even after significant structural deformation.

Heart models were produced with a TPU using increasing energy values ranging from 0.16 J/mm^3^ to 0.30 J/mm^3^. Surgeons qualitatively assessed the flexibility of these heart models.

We selected the optimal parameters to meet the qualitative criteria for flexibility and thinness of 0.5 mm: a single contour with a laser power of 14 W (scan speed Ss = 3000 mm/s and Ed = 0.16 J/mm^3^). However, excessive rigidity for handling was observed when energy levels exceeded 0.25 J/mm^3^.

Shape memory was tested by comparing the models before and after stretching. After subjecting it to 50 cycles of full deformation, we scanned the surface and compared it to the original model. On average, there was a variation of 0.036 ± 0.4 mm compared to the original model (see figure in the Supplementary Data).

**Figure 6:**
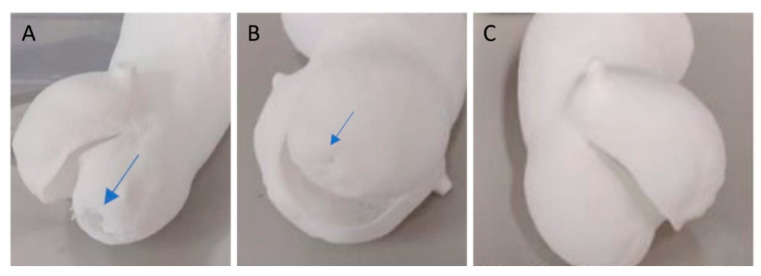
Model of an aortic valve with a simple contour. (**A**) A 0.3 mm thickness valve using a low-energy technique (preset TPU-2 with Ed = 0.21 J/mm^3^) and (**B**) a 0.4 mm thickness valve both exhibit small holes (indicated by the blue arrow) in the thin printed section. In comparison (**C**), the 0.5 mm thickness TPU model is flawless.

When using a simple contour laser scan at low energy density for aortic valves, a thickness of at least 0.5 mm is needed. Thinner sections might develop holes during processing, especially in flat areas, likely due to reduced resistance to tearing (Figure 6).

**Figure 7:**
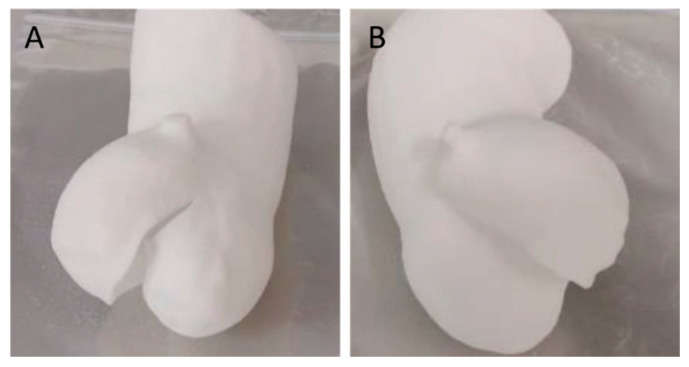
Additive manufacturing of a TPU 0.3 mm thick bicuspid aortic valve using an additional reinforcement scan layer using a laser power of 12 W (preset TPU-1). Double contour (**A**) and edge (**B**) enable successful manufacturing.

By employing the ‘double contour’ or ‘edge’ laser scan patterns, we were able to successfully create bicuspid aortic valve structures with thicknesses of 0.4 mm and 0.3 mm using a laser power of 12 W. These patterns reinforced the thin structure, providing it with the necessary stiffness and flexibility (Figure 7).

**Figure 8:**
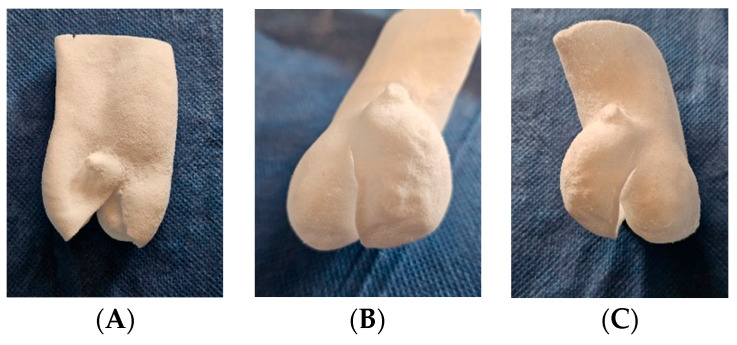
Additive manufacturing of bicuspid valve TPE models with thicknesses of (**A**) 0.5 mm, (**B**) 0.8 mm, and (**C**) 1.3 mm, obtained with the TPE-7 preset of 14 W and laser speed of 30,000 dps (1800 mm/s). (**A**) A 0.5 mm thick model: loss of shape memory with sagging. (**B**) A 0.8 mm thick model: flexible and resistant to handling. (**C**) A 1.3 mm thick model: retains adequate flexibility with greater strength.

The TPE-7 preset was optimal, selected at 14 W and Ss = 30,000 dps (1800 mm/s), resulting in an Ed = 0.648 J/mm^3^. This configuration yielded a tensile strength of 0.91 ± 0.05 MPa, elongation of 161.50 ± 11.26%, and an E modulus of 2.18 ± 0.19 MPa. However, using different laser powers, we faced challenges in achieving parts with an E exceeding 4 MPa, even with high energy densities (ranging from 1.06 to 1.4 J/mm^3^). This limitation may be attributed to factors such as the laser spot size and control of powder temperature within the printer. However, when comparing models obtained with the same preset and thickness of 0.5mm, 0.8mm, and 1.3mm, shape memory is not retained in the 0.5mm model (Figure 8).

#### 3.4.2. Ultrasound Imaging Properties of the TPU Models

**Figure 9:**
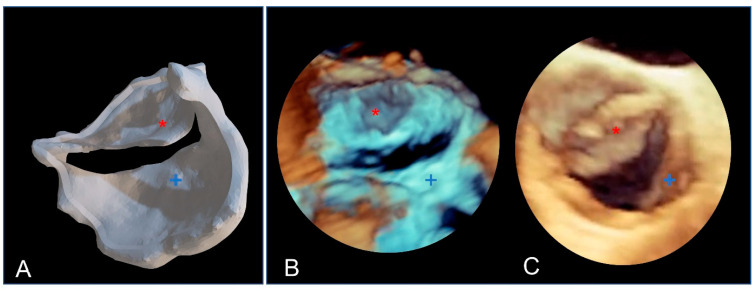
The bicuspid aortic valve in a similar face-plan view. (**A**) View of the 3D printed model. (**B**) A 3D ultrasound view of the immersed model (with two thin aortic cusps marked with * and +). (**C**) A 3D ultrasound in situ view of the same valve in the patient, showing both cusps (+ and *).

**Figure 10:**
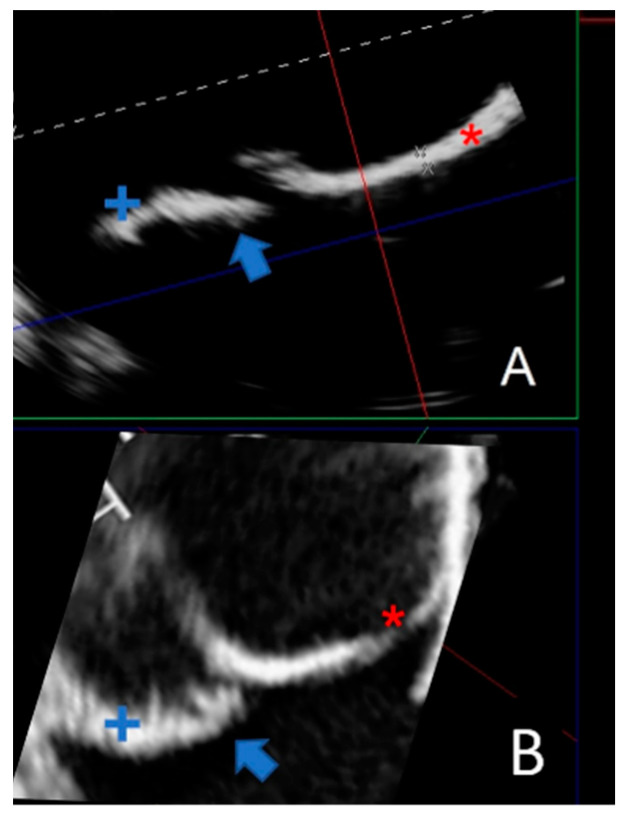
Comparing a 2D echographic view of the bicuspid aorta model to the in situ view. (**A**) A 2D ultrasonograph of an immersed bicuspid aorta model with two cusps (+ and *) and prolapse of one cusp (blue arrow). (**B**). A 2D in situ ultrasound in the same orientation as A, with two cusps (+ and *) and cusp prolapse (blue arrow).

Due to their porous structure, the TPU and TPE models exhibit interesting echogenic properties. We compared the echogenic rendering with in situ images of the same valve (refer to Figure 9 and Figure 10). The valve structure was accurately depicted in both 3D and 2D views. Notably, the cusp in situ thickness measurements align with the model’s 0.3 mm thickness.

## 4. Discussion

### 4.1. Modulating the Mechanical Properties of TPU and TPE through Exposure Parameters

In this study, we investigated the potential for customizing the mechanical properties of components within a broad range, adjusting the exposure parameters (Hs, Ss, Lp, Ed) to achieve an E modulus ranging from 28 MPa to 71 MPa. The energy density parameter relates to laser power and laser scan speed. Increasing the laser power and decreasing laser scan speed leads to an increase in energy density [30]. So, the tensile strength and elongation at break decrease as the scanning speed increases or the laser power drops.

The scanning speed determines the duration of exposure time for powders to be sintered; the laser power indicates the inputted thermal energy onto the powder bed in unit time.

Increasing energy density leads to better powder coalescence (parts with lower porosity) and, thus, to optimal mechanical properties. The tensile strength considerably decreases when the scanning speed increases; therefore, the lower scanning speed is preferable to control the coalescence among powders. If the energy density is too low (lower laser power and higher laser scan speed), part porosity may increase with decreased mechanical properties.

Conversely, exceeding a certain threshold in laser power (or having too low a laser scan speed) results in a decline in mechanical properties due to thermal degradation of the polymer (Figure 3 middle), especially noticeable with TPE exposure parameters (Figure 5b).

These findings are consistent with previous research conducted by Yuan et al. [31], who observed a 2.5-fold increase in tensile strength (from 2 MPa to 6.5 MPa) as the laser scan speed decreased (from 4000 to 3000 mm/s) with a constant energy density (ED) of 0.20 J/mm^3^ to 0.27 J/mm^3^, a layer thickness (Hs) of 0.1 mm, and a laser power of 8 W using an EOS P395 system. This trend remains similar for increasing laser powers up to 14 W and delivered energies of 0.47 W and 0.35 W, resulting in a UTS of 14 MPa and 7 MPa, respectively.

In a similar vein, Verbelen et al. [32] achieved comparable properties with a fixed Hs of 0.1 mm, where the ultimate tensile strength (UTS) increased from 6.5 MPa and elongation at break increased from 6.5 MPa and 350% at LP = 8 W with ED = 0.27 J/mm^3^ to 14 MPa and 500%, respectively, at LP = 14 W with ED = 0.47 J/mm^3^.

Dadbakhsh [33] utilized lower energy density values with a DTM Sinterstation 2000 machine and a TPU powder with a D50 of 63 microns, obtaining a comparable UTS of 3.8 MPa and an elongation at break of 277% at 0.14 J/mm^3^ and a UTS of 18 MPa and an elongation at break of 559% at 0.67 J/mm^3^ (laser = 10 W, speed = 1000 mm/s and a similar layer thickness of 0.1 mm but a fixed Hs = 0.15 mm)

In this study, our primary goal was to achieve maximum flexibility by minimizing the exposure energy to 0.16 J/mm^3^, resulting in an ultimate tensile strength (UTS) of 3.28 MPa.

Beyond energy delivery, we specifically studied the parameter Hs. Scanning line spacing (Hs) is not frequently investigated in the literature for TPU polymers. Our study utilized two Hs values ranging from 0.2 mm to 0.25 mm, which is broader than the typical 0.1 mm range. Decreasing Hs from 0.25 to 0.20 mm resulted in a notable alteration in Young’s modulus, increasing by almost 50% from 38 MPa to 62 MPa. Thus, hatching distance significantly affects the mechanical properties of TPU. When the hatching distance is decreased, the laser beam overlay increases (and the energy density). The increase in the laser beam overlay leads to a local rise in temperature (which decreases the polymer viscosity) and to a denser part, thus improving inter-layer cohesion. This explains why decreasing (at least to some extent) the Hs leads to an increase in Young’s modulus of parts when comparing samples with the same energy density but a higher Hs.

Pilipović et al. [34] demonstrated the significance of the d/Hs overlap ratio (where d is the laser beam diameter) when using Formiga P100 EOS equipment on PA12. Specifically, they emphasized that when Hs is less than the laser beam diameter, maximum values of tensile properties can be achieved.

Nevertheless, our current study reveals that, for TPU, the positive impact linked to the reduction in Hs is amplified and surpasses the overlap factor. We observe an almost twofold increase in tensile strength with a relative decrease in Hs from 0.25 to 0.2 mm.

Optimizing parameters in additive manufacturing, mainly with selective laser sintering, is complex and relies on various factors, both internal (chemical, thermal, and rheological) [35,36] and external (type of equipment, laser characteristics: type and focal width, and temperature) [28], making the optimization process challenging.

Our study found that considering all the exposure parameters (Hs, Ss, LP, and Ed) allows for accurate prediction of the elasticity modulus (E), with a high R^2^ value of 0.977. We can predict E with less than a 2 MPa error (see additional data in the appendix), covering a range from 28 MPa to 71 MPa. However, when only energy density is considered, the correlation coefficient drops to R^2^ = 0.64, resulting in a more significant standard deviation of 8 MPa in the predicted E. This highlights the considerable impact of each parameter on mechanical properties beyond their contribution to overall energy density.

Furthermore, the influence of spatial orientation on mechanical properties, as demonstrated by Goodridge et al. [37], reveals a significant dependence, varying by about 25 to 50%. According to their findings, parts printed in the Z direction exhibit the lowest tensile strength, while those in the X direction display the highest tensile strength.

Our results align with those of Tao Xu, who similarly observed lower mechanical strength in the upright direction [38]. This highlights the significance of taking into account the anisotropic tensile properties of selectively laser-sintered TPUs and TPEs in the manufactured structures [39].

In our aortic models, another factor comes into play: rugosity. While not evident in TPUs, as SEM sections show a dense structure, it becomes more pronounced in TPEs, mainly on flat surfaces.

While the optimal TPE preset selected exhibits a dense center, it still shows peripheral rugosity, as illustrated by the SEM sections (Figure 5b left). This phenomenon can be attributed to the thermal gradient, especially considering our work at room temperature. When the laser scans the surface in thin layers, it may melt particles on the sides, leading to small bumps or defects on the surface. This characteristic affects TPE prototypes, resulting in a grainy surface, while TPU prototypes remain smooth.

Increasing Ed (energy density) densifies the part but leads to central degradation (refer to Figure 5b right) due to a rise in local temperature. Working in an inert atmosphere, such as nitrogen, could potentially mitigate this effect.

### 4.2. Application to the Manufacture of Heart Valve Structures

Achieving a membrane thickness in the submillimeter range is crucial in tissue-engineered heart valves and presents notable challenges. One of the primary challenges is maintaining their resilience to dynamic stress.

The thickness of a normal aortic cusp is approximately 0.3 to 0.8 mm [40]. The Young’s modulus of native aortic valves, mainly dependent on collagen fibers, is anisotropic, approximately 11.91 ± 7.18 MPa in the circumferential direction (vs. 1.3 MPa in the radial direction) during the diastolic phase. The ultimate strain at break for valve leaflets is approximately 30% in the radial direction and approximately 20% in the circumferential direction [41]. This is essential to prevent excessive leaflet bulging or prolapse due to transvalvular pressure [42].

The elastic modulus of the aortic aneurysm is around 2.3 ± 1.6 MPa, with a thickness of 1.97 mm ± 0.3 and a rupture resistance of 1.26 MPa ± 0.9 MPa [43].

The TPU-SLS technique can print anatomical aortic parts with 0.5 mm thick leaflets using a single-contour structure with an E modulus of 20 MPa. A double contour or edge could reduce the thickness to 0.4 mm or even 0.3 mm, enhancing leaflet flexibility.

Using TPE 210-S (ALM^®^) on the TPE SnowWhite printer, we found an optimal energy delivery of Ed = 0.648 J/mm^3^, resulting in an E modulus of 2 MPa, similar to the aorta in situ. Those mechanical properties align with previous studies using TPUs such as Lubrizol Estane^®^ for valvular scaffolds [44] via melt-molding techniques.

Although compression molding [23] or electro-spraying [24] and electrospinning [25] are commonly chosen methods for valve scaffolding, they may come with inherent risks. These include difficulties separating the polymeric heart valve structure from the mold, particularly with thin leaflets, the possibility of air microbubble insertion if speed settings are incorrect, and the critical challenge of rapidly cooling molten polymers. For instance, Atari et al. produced 3D valve scaffolds using molding and based on an elastomeric poly-glycerol sebacate polymer, which required a cusp thickness of 1.2 mm [45].

Hockaday et al. utilized extrusion (FDM) printing to fabricate aortic valve scaffolds with a macromer that polymerizes via radical crosslinking. Models were based on CT scans of fixed porcine aortic valves, and shape fidelity was found to be around 70% for smaller structures [46].

In comparison, selective laser sintering (SLS) with TPUs and TPEs effectively overcomes these challenges. The comparison of the 3D printed model by SLS with the STL file derived from the CT scan was excellent, with a difference of 0.26 mm ± 0.57 mm (more data in additional file).

One key objective in TEHV research is to explore diverse geometric structures for optimizing pressure flow and leaflet shear stress. In a recent study, Schröter et al. utilized an inkjet 3D printer to test and validate a multi-triangular leaflet shape fabricated in silicone. However, the leaflet thickness ranged from 1 mm to 3 mm [47]. Utilizing 3D printing via the SLS process, we can produce diverse valvular scaffolds reproducibly, featuring thin membranes up to 300 µm that are flexible, tear-proof, and exhibit adequate resistance to handling and shape memory. This method offers an advantage over other printing methods by eliminating the need to remove residual material.

Furthermore, the high shape memory capability of TPUs [48] is preserved even for structures as thin as 0.3 mm in our study, which is crucial for fabricating valve scaffolds.

Davood Rahmatabadi et al. demonstrated that by incorporating just 30% TPU (thermoplastic polyurethane) with 70% PLA (polylactic acid) in a composite manufactured through fused deposition modeling 3D printing, they significantly improved the shape memory performance [49]. Their composite displayed shape fixity and shape recovery rates of up to 100% and 91%, respectively. Employing a Box–Behnken response surface methodology, they concluded that infill density significantly impacted shape memory properties. This aligns with our findings regarding the application of additional contours (double contour or edge), which precisely acts to increase the infill density [50].

The benefits of aortic valve models in aortic valve surgery can be twofold:

First, they can be used as simulation models for surgical interventions. This was the primary aim of our aortic bicuspid models. Surgeons can enhance their skills by practicing on these models, which replicate the flexibility of tissues [29].

Sometimes, the aortic valve, which typically has three leaflets, can become bicuspid, with only two leaflets. This change increases the risk of leakage between the leaflets [51]. Surgical correction involves folding and repairing the leaflets to restore a proper seal between them [52], a procedure known as aortic plasty [53]. However, this operation requires skill and experience, and success often depends on the surgeon’s learning curve [54].

In this context, the flexible 3D anatomical model can be of great help for preoperative valve analysis, preoperative planning, surgical training, and safe model handling [55].

In the same field, we highlight a potential application in three-dimensional echocardiography due to the echogenicity of TPU parts. When immersed and explored with a 3D ultrasound probe, TPU parts closely resemble the volume rendering observed in situ. As a result, they can be integrated into precise training in three-dimensional echocardiography [56].

The critical acoustic properties of materials, like sound velocity and impedance, depend on their density and structure. When using techniques like SLS for 3D model production, materials like TPUs and TPEs become echogenic due to micro-porosities, making them suitable for applications like echocardiography training [57].

We demonstrate excellent ultrasound reflection without loss, dispersion, or absorption, allowing accurate ultrasound 3D rendering even in samples with a thickness as low as 0.3 mm, which closely resembles the original STL model. In various directions, measurements of wall thickness and dimensions show no image distortion compared to the original STL file (additional data and figures are provided in the Supplementary Materials).

### 4.3. Future Vision

Our findings may apply to polymeric heart valves made from synthetic materials to combine the favorable flow dynamics of biological valves with the durability of mechanical ones.

This idea, dating back to early heart valve prosthetics, has seen renewed interest due to advances in polymer science [58].

The field of all-polymer valves is continuously advancing, with research focusing on enhancing their physical and mechanical properties and their durability against degeneration, calcification, or thrombogenicity.

Various polymers have been explored for this purpose: elastomeric poly-glycerol sebacate polymer, poly E-caprolactone [45], polystyrene-block-isobutylene-blockstyrene [59], and polyurethane copolymers [23].

Polyurethanes have already been utilized in heart assist devices, with examples including Angioflex^®^ (a proprietary PU developed by ABIOMED Inc.) [60]. Advanced prototypes for aortic [24] and mitral valves [15] have also been proposed.

While molding is the technique used for mass production, 3D printing is an essential option in producing and testing prototypes [58]. Research and development on novel valve geometry concepts have recently been proposed using additive manufacturing [47]. In prototyping valvular scaffolds, the SLS technique can find a significant place due to its spatial resolution, finesse, smoothness of the produced parts, and ability to reproduce complex shapes.

Beyond the macrostructure, tissue-engineered heart valve research increasingly focuses on complex bioinspired microstructures that mimic the tri-layered arrangement of aortic leaflets and the specific orientation of collagen and fibrin fibers.

For example, Voulter et al. [26] proposed a valvular scaffold derived from CT scan anatomy using additive techniques and molding.

They utilized molding combined with a spraying method of a photo-curable silicone to create leaflets with tunable mechanical properties. Additionally, reinforcement fibers were superimposed using direct ink writing in a selected transverse direction to enhance the structure. To provide support, an over-molded cap matching the aorta geometry was employed, along with a hexachiral auxetic pattern ink written to form the stent-like structure.

Similarly, utilizing functionally graded materials, which exhibit gradual changes in desired directions and specific mechanical properties, shows promise, as well as anisotropic 3D printing through photo-curing 3D printing using digital light processing, an accurate method that employs photosensitive resin [61].

Electrospinning is another highly versatile and efficient fabrication technique that can control the alignment of electrospun microfibers to create mechanical anisotropy [25]. Therefore, electrospun scaffolds of polyester urethane have been manipulated to achieve specific bending stiffness for heart valve tissue engineering [62].

The second promising application is the use of biocompatible, non-thrombogenic, and non-immunogenic membranes, which are 3D-shaped and customized membranes to fit specific anatomies for in situ use in aortic valve surgery, rather than solely focusing on the entire valve [63]. TPUs’ strong suture retention^13^ makes them even more suitable for surgical aortic substitutes.

In procedures like the Ozaki technique [64] for repairing aortic leaflets, particularly in young patients, autologous pericardium or PTFE is used [65]. However, these materials are challenging to shape can calcify and harden over time [66]. Thus, soft, biocompatible, SLS 3D-shaped, and personalized membranes might be a key.

While recognizing that a single polymer (whether natural or synthetic) may not fulfill all requirements for TEHVs, including mechanical properties, dynamic resilience, and biological properties, the advantageous properties of TPUs tuned with the selective laser sintering process make them suitable for creating valvular models.

## 5. Limitations

The main limitation of this study is that the industry did not disclose the exact structure of TPU 1301 (EOS^®^ GmbH, Munich, Germany). However, it is known that, like all TPUs, the chemical composition of polyurethanes consists of three main components: a polyether, polyester, or polycarbonate diol; a chain extender (low-molecular-weight diol); and a diisocyanate.

Although we only conducted uniaxial testing, there is a need for further exploration of additional mechanical properties, such as resilience to cyclic mechanical stress, flexural-bending behavior, and in vitro fatigue testing. Furthermore, in vivo tests for biocompatibility and thrombogenicity should also be performed [67].

Another aspect to consider is the anisotropy of mechanical properties along the x, y, and z axes inherent to the SLS technique, which differs from other methods like molding. However, this phenomenon was more noticeable with TPE than TPU, especially considering the energy range delivered. Furthermore, this challenge can be addressed by integrating transverse microbands into geometric structures, replicating the arrangement of collagen fibers in the cusps.

While we have identified an optimal preset for the valvular model, future research could explore using different SLS laser exposure presets within the same scaffold. This would enable the customization of rigidity levels for various valve segments, including the annulus, leaflets, or attachment points.

Regarding TPEs, one limitation lies in increasing smoothness through higher laser exposure without compromising the material’s integrity. One potential solution is utilizing an SLS machine in an inert gas environment like nitrogen. The significant roughness observed with TPE could be a limiting factor since it affects platelet adhesion, thus impacting biocompatibility [68]. Therefore, addressing surface roughness may be essential to enhance the biocompatibility of SLS-TPE-based structures.

## 6. Conclusions

The SLS-TPU/TPE technique demonstrates the feasibility of producing a fine valve structure with a unique capability among additive manufacturing techniques—producing soft and resistant micrometric membranes. Controlling and modulating SLS exposure parameters makes it possible to vary mechanical properties and modulate the flexibility–strength balance precisely.

The TPU and TPE-SLS techniques are well suited for producing shape memory aortic valve models that reproduce strength, flexibility, and fineness, making them particularly suitable for surgical training and handling. Additionally, they exhibit exciting echogenicity properties. Moreover, their potential biocompatibility opens a wide field for future studies on customized patient-specific 3D-manufactured valve substitutes.

## Figures and Tables

**Table 1:** Data from differential scanning calorimetry (DSC) curves. T_ic_: onset of crystallization temperature; T_if_: onset melting temperature; T_c_: peak of crystallization, T_f_: peak of melting temperature; ΔT: difference T_if_–T_ic_.

Sample	T_ic_ (°C)	T_if_ (°C)	ΔT	T_c_ (°C)	T_f_ (°C)
TPU	117.7	121.4	3.7	109.5	130.6

**Table 2:** Laser exposure parameters.

TPU	Hatching Distance (Hs-mm)	Laser Scan Speed (Ss-mm/s)	Laser Power (W)	Energy Density (J/mm^3^)
1	0.25	3000	12	0.16
2	0.25	3000	16	0.21
3	0.25	3000	18	0.24
4	0.25	3000	19	0.25
5	0,25	3000	25	0.33
6	0.25	2500	16	0.26
7	0.20	3000	16	0.27
8	0.25	3000	22	0.29
9	0.25	2500	19	0.30
10	0.20	3000	19	0.32
11	0.20	2500	16	0.32
12	0.25	2500	22	0.35
13	0.20	3000	22	0.37
14	0.20	2500	19	0.38
15	0.25	2000	16	0.32
16	0.25	2000	19	0.38
17	0.25	2500	25	0.40
18	0.25	2000	22	0.44
19	0.25	2000	25	0.50

**Table 3:** Exposure parameters and corresponding mechanical properties of TPU standard samples. (ultimate tensile strength (UTS) and elastic modulus (E), hatching distance (Hs), laser scan speed (Ss), laser power (LP), energy density (Ed), elongation at break (dL_(rupture)_), elongation at maximal strength (dL(F_max_)).

TPU	Hs- mm	Ss-mm/s	LP- W	Ed J/mm^3^	UTS MPa	dL(F_max_) %	Strength at Break	dL_(rupture)_ %	Module E MPa
1	0.25	3000	12	0.16	3.28 ± 0.14	132.37 ± 10.82	3.20 ± 0.17	138.74 ± 9.61	28.23 ± 0.81
2	0.25	3000	16	0.21	5.22 ± 0.13	169.04 ± 4.55	4.98 ± 0.19	177.70 ± 5.67	47.27 ± 1.96
3	0.25	3000	18	0.24	5.61 ± 0.08	188.11 ± 9.45	5.35 ± 0.19	195.84 ± 12.20	52.07 ± 1.50
4	0.25	3000	19	0.25	5.07 ± 0.36	119.54 ± 42.61	4.88 ± 0.25	123.07 ± 45.20	53.93 ± 2.32
5	0.25	2500	16	0.26	4.48 ± 0.18	61.81 ± 13.54	4.40 ± 0.05	62.94 ± 13.93	55.78 ± 2.79
6	0.20	3000	16	0.27	7.19 ± 0.21	271.81 ± 10.78	6.87 ± 0.22	281.52 ± 10.67	63.35 ± 5.33
7	0.25	3000	22	0.29	5.90 ± 0.16	133.07 ± 15.80	5.73 ± 0.16	134.98 ± 15.91	62.89 ± 1.13
8	0.25	2500	19	0.30	5.61 ± 0.33	99.83 ± 18.66	5.50 ± 0.37	101.54 ± 18.25	60.65 ± 4.28
9	0.20	2500	16	0.32	7.88 ± 0.16	334.40 ± 10.76	7.50 ± 0.14	344.28 ± 10.79	72.31 ± 8.39
10	0.20	3000	19	0.32	7.50 ± 0.20	318.90 ± 14.52	7.26 ± 0.22	332.32 ± 18.21	66.28 ± 2.57
11	0.25	2000	16	0.32	4.99 ± 0.13	163.93 ± 7.24	4.83 ± 0.22	170.58 ± 8.30	57.87 ± 2.63
12	0.25	3000	25	0.33	7.07 ± 0.05	246.61 ± 8.08	6.76 ± 0.15	254.24 ± 11.37	70.26 ± 1.87
13	0.25	2500	22	0.35	6.47 ± 0.19	230.60 ± 32.43	6.17 ± 0.20	239.14 ± 32.27	67.65 ± 3.86
14	0.20	3000	22	0.37	8.43 ± 0.25	380.06 ± 27.16	8.07 ± 0.42	390.41 ± 25.52	74.17 ± 2.60
15	0.20	2500	19	0.38	8.69 ± 0.15	394.36 ± 8.35	8.40 ± 0.17	405.02 ± 10.19	75.85 ± 3.74
16	0.25	2000	19	0.38	5.78 ± 0.29	189.55 ± 17.50	5.59 ± 0.27	197.90 ± 18.35	63.20 ± 1.99
17	0.25	2500	25	0.40	6.80 ± 0.08	245.41 ± 7.71	6.58 ± 0.17	251.73 ± 7.22	72.47 ± 3.64
18	0.25	2000	22	0.44	6.17 ± 0.04	205.73 ± 5.60	5.93 ± 0.14	217.20 ± 8.73	66.75 ± 1.96
19	0.25	2000	25	0.50	6.17 ± 0.08	177.60 ± 7.55	6.01 ± 0.13	183.75 ± 8.14	71.28 ± 1.12

**Table 4:** Uniaxial tensile strength of TPU samples of 0.5 mm thickness according to exposure parameters, spatial orientations (45°, upright, on edge) and laser scan mode: simple, double contour, edge (abbreviations as in Table 3).

	UTS (MPa)	dL(F_max_)	E (MPa)
Simple contour 14 w (45°)	1.85 ± 0.16	29.71 ± 5.68	22.55 ± 1.73
Simple contour 14 w (on edge)	3.44 ± 0.20	88.40 ± 8.21	27.88 ± 1.27
Simple contour 14 w (upright)	1.62 ± 0.24	25.40 ± 5.77	20.85 ± 2.76
Simple contour 16 w (45°)	2.61 ± 0.09	29.71 ± 5.68	22.55 ± 0.87
Simple contour 16 w (on edge)	4.47 ± 0.07	127.91 ± 6.41	32.06 ± 0.74
Simple contour 16 w (upright)	2.03 ± 0.20	34.55 ± 3.40	22.97 ± 2.00
Edge 12 w (45°)	4.03 ± 0.37	61.21 ± 16.78	40.29 ± 0.40
Edge 12 w (on edge)	5.66 ± 0.69	148.05 ± 16.74	40.41 ± 3.90
Edge 12 w (upright)	3.27 ± 0.20	41.82 ± 6.12	36.38 ± 1.19
Double contour 12 w (45°)	4.92 ± 0.17	53.61 ± 4.96	50.86 ± 0.74
Double contour 12 w (on edge)	7.13 ± 0.51	150.53 ± 16.41	50.02 ± 2.34
Double contour 12 w (upright)	4.26 ± 0.38	40.91 ± 3.05	47.91 ± 2.77

**Table 5:** TPE exposure characteristic sets and their corresponding mechanical properties. (Ultimate tensile strength (UTS) and elastic modulus (E), hatching distance (Hs), laser scan speed (Ss) points per second, where a point corresponds to 60 microns, laser power (LP), energy density (Ed), elongation at break (dL_(rupture)_), elongation at maximal strength (dL(F_max_))).

TPE	% Laser 14 W	% Edge	Ss Laser dps	Ed (J/mm^3^)	UTS (MPa)	dL(F_max_) %	Strength at Break (MPa)	dL_(rupture)_ %	Modulus E (MPa)
1	70	65	10,000	1.36	1.06 ± 0.20	147.27 ± 57.24	0.94 ± 0.23	156.78 ± 58.77	4.10 ± 0.36
2	70	65	40,000	0.340	0.42 ± 0.03	117.01 ± 5.91	0.41 ± 0.04	119.01 ± 5.48	0.74 ± 0.04
3	70	65	45,000	0.302	0.43 ± 0.03	117.28 ± 5.98	0.43 ± 0.03	118.71 ± 5.53	0.87 ± 0.06
4	70	65	50,000	0.272	0.30 ± 0.02	100.41 ± 4.57	0.29 ± 0.02	102.82 ± 4.26	0.52 ± 0.04
5	100	95	10,000	1.94	1.40 ± 0.16	262.94 ± 44.35	1.35 ± 0.16	276.89 ± 45.97	4.59 ± 0.32
6	100	95	25,000	0.78	0.66 ± 0.01	85.19 ± 7.37	0.65 ± 0.01	93.88 ± 19.54	2.31 ± 0.26
7	100	95	30,000	0.648	0.93 ± 0.05	157.64 ± 10.89	0.91 ± 0.05	161.50 ± 11.26	2.18 ± 0.19
8	100	95	40,000	0.49	0.37 ± 0.06	96.54 ± 17.34	0.36 ± 0.06	101.83 ± 13.66	0.75 ± 0.07

## Data Availability

CIOBOTARU, VLAD (2023), “Modelling Mechanical Properties of Thermoplastic Polyurethanes through Laser Sintering Exposure for Replicating Micrometric Aortic Valve Membranes”, Mendeley Data, V1, doi:10.17632/wfsm6f9rbn.1.

## Supplementary Materials

The following supporting information can be downloaded at: https://www.mdpi.com/article/10.3390/polym16070900/s1.

## Abbreviations

AM: additive manufacturing; TPU: thermoplastic polyurethane; TPE: thermoplastic elastomers; SLS: selective laser sintering; Hs: hatching distance; E_D_: energy density; UTS: ultimate tensile stretch.
