# Supplementary material for: Aortic Valve Engineering Advancements: Precision Tuning with Laser Sintering Additive Manufacturing of TPU/TPE Submillimeter Membranes
Source: Polymers (Basel). 2024 Mar 25;16(7):900. doi: 10.3390/polym16070900 (PMC11013727; doi:10.3390/polym16070900)

Table S1.

1.1: Correlation Matrix

	Hs	Ss	W	Ed	Modulus E
Hs	**1**	-0,257	0,164	-0,086	-0,438
Ss	-0,257	**1**	-0,187	-0,677	-0,311
W	0,164	-0,187	**1**	0,747	0,700
Ed	-0,086	-0,677	0,747	**1**	0,802
Modulus E	-0,438	-0,311	0,700	0,802	**1**

1.2: Regression of Variable “E”: Regression Coefficients “E”

Observations	19
Somme des poids	19
DDL	14
R^2	0,977
R^2 ajusté	0,970
MCE	3,886
RMCE	1,971
MAPE	2,671
DW	1,673
Cp	5,000
AIC	29,990
SBC	34,712
PC	0,039

Variable	Observations	Obs. avec données manquantes	Obs. sans données manquantes	Minimum	Maximum	Moyenne	Ecart-type
E	19	0	19	28,234	75,845	62,225	11,461
Hs	19	0	19	0,200	0,250	0,237	0,023
Ss	19	0	19	2000,000	3000,000	2631,579	402,841
W	19	0	19	12,000	25,000	19,368	3,624
Energie	19	0	19	0,160	0,500	0,321	0,082

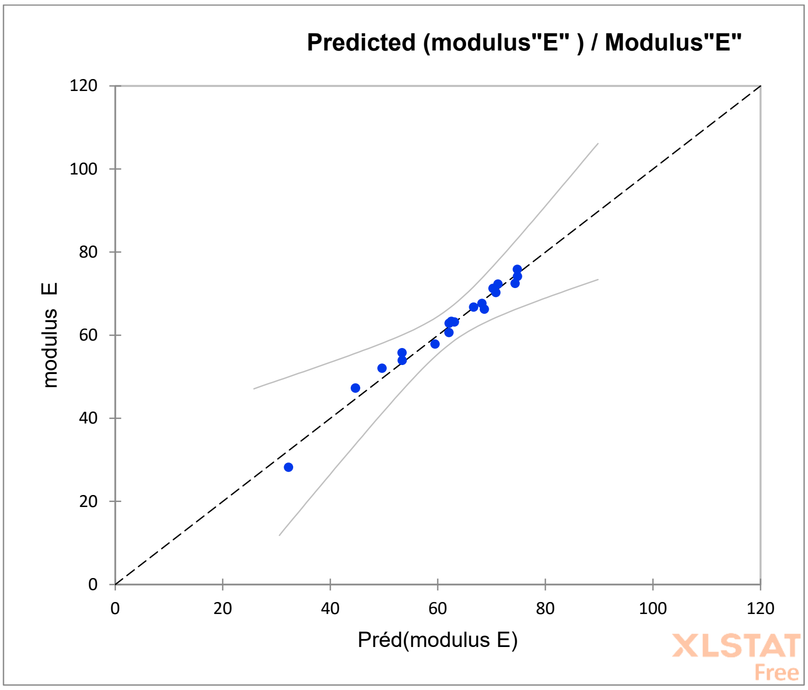

1.3: Normalized residues / Modulus E

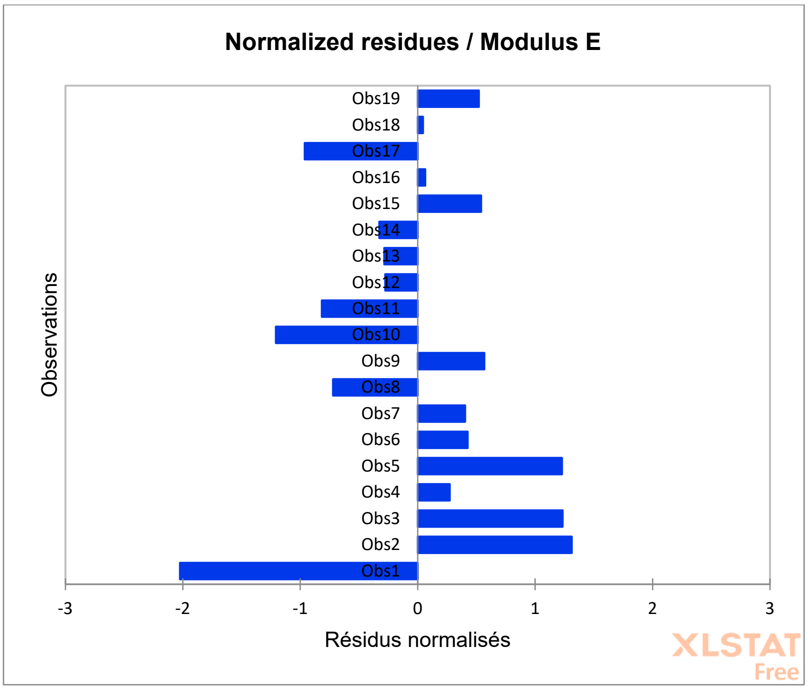

E(Mpa) = 292,006792120481-663,395439304767*Hs(mm)-4,29180303911739E-02*Ss(mm/s)+6,31100628369743*LP(W)-255,686834505214*Ed(j/mm3)

Figure S1: Reproducibility: 3D comparisons, of scanning prints obtained from replicas using the same presets, particularly focusing on the aortic leaflets. The average distance was 0.04mm +/-0.3mm (see figure).

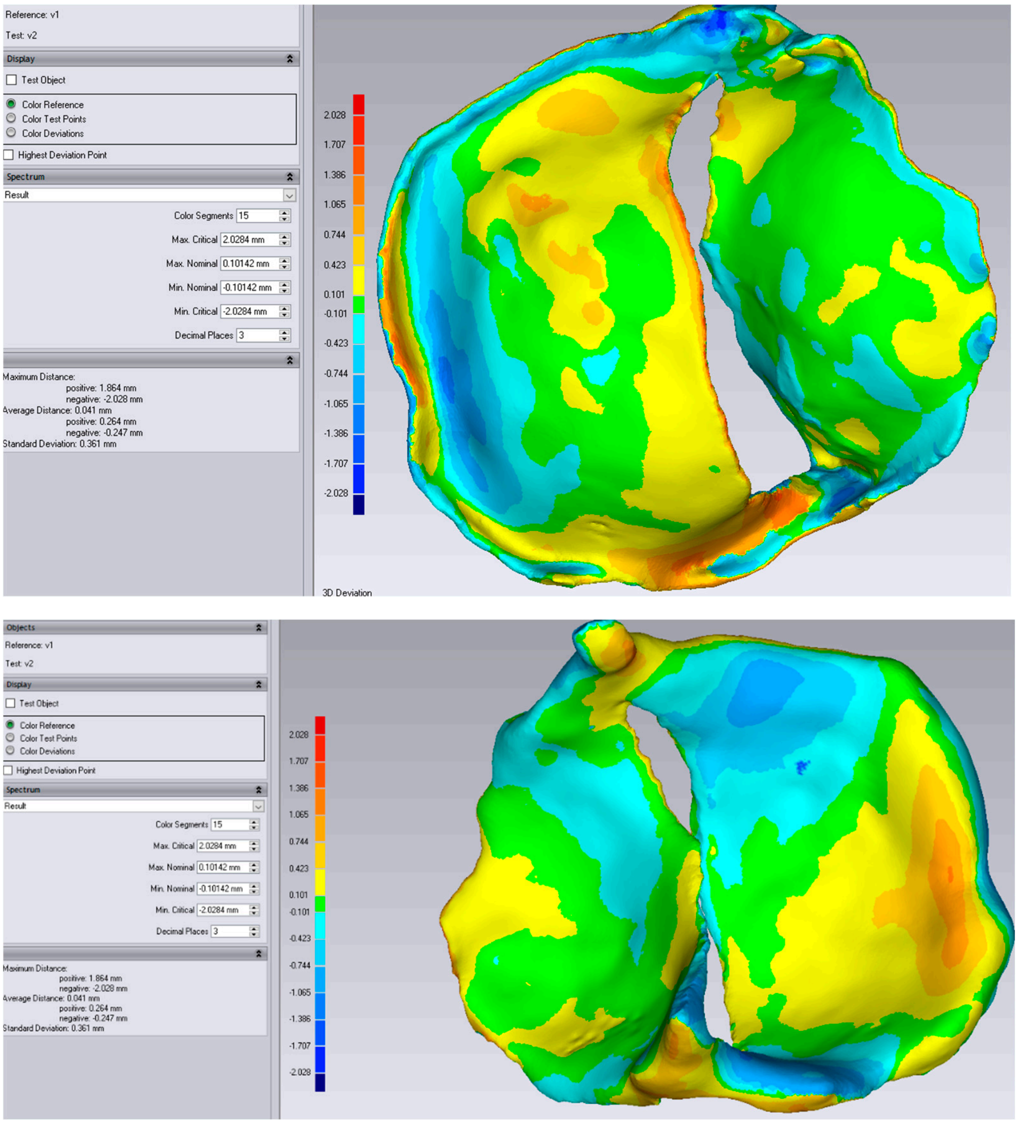

Figure S2: Shape memory: comparison study of the models after stretching. Scan of the surface of the piece immediately after 50 cycles of full deformation and compared it to the initial STL file of the model. The average variation was 0.036 ± 0.4mm relative to the base STL model.

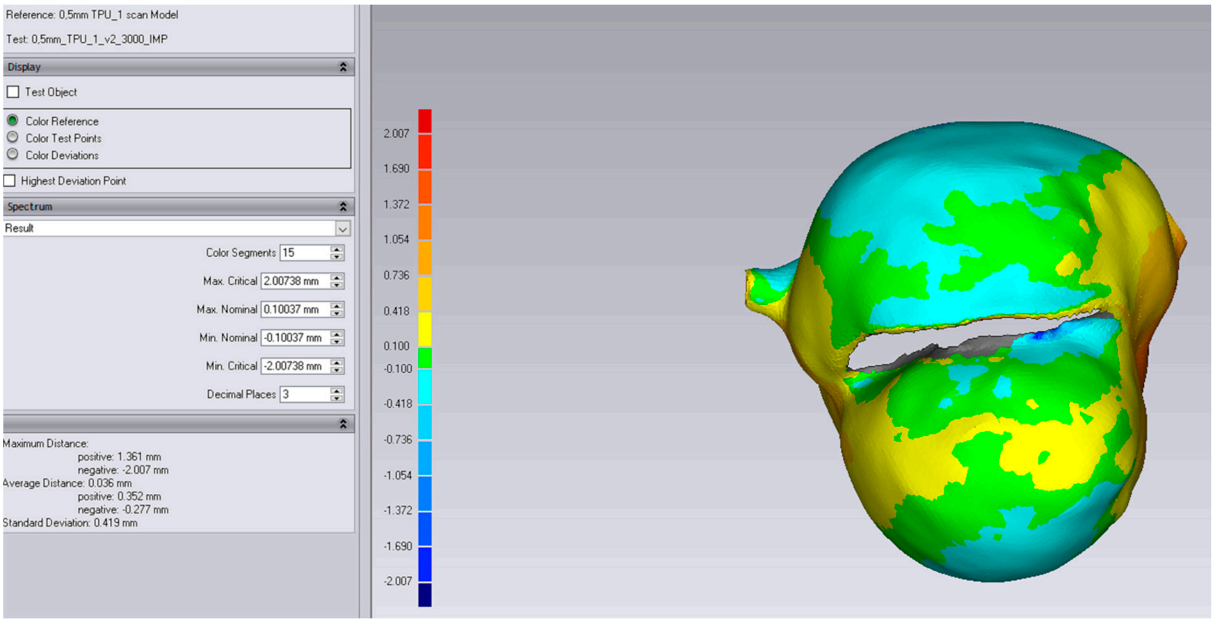

Figure S3: Comparison between the stl file obtained from the cardiac computer tomography and the 3d printed model.

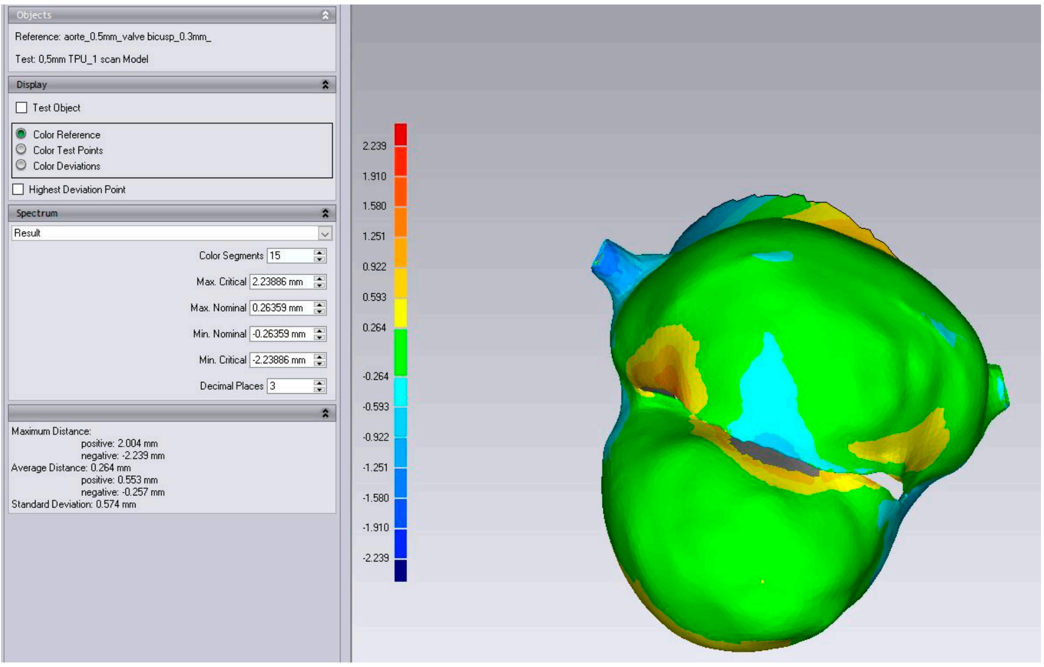

Figure S4: Ultrasound 3D Rendering: Achieving High Fidelity in Samples as Thin as 0.3mm, Resembling Original STL Models

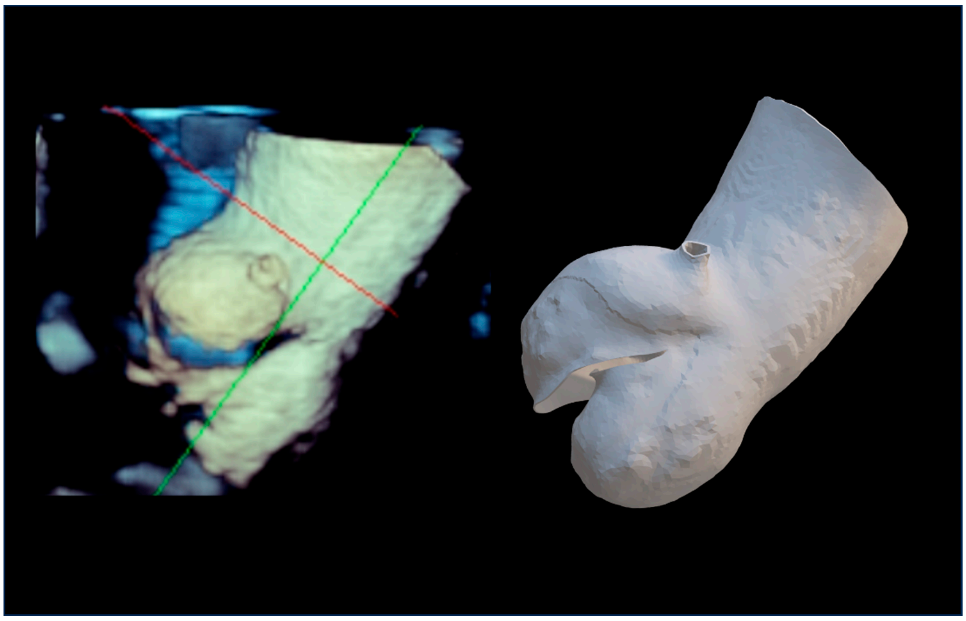

Figure S5: Aortic Leaflet Thickness Measurement: Ultrasound Evaluation of 0.06cm with Equivalent Thickness of 0.05cm in a 3D Printed Model

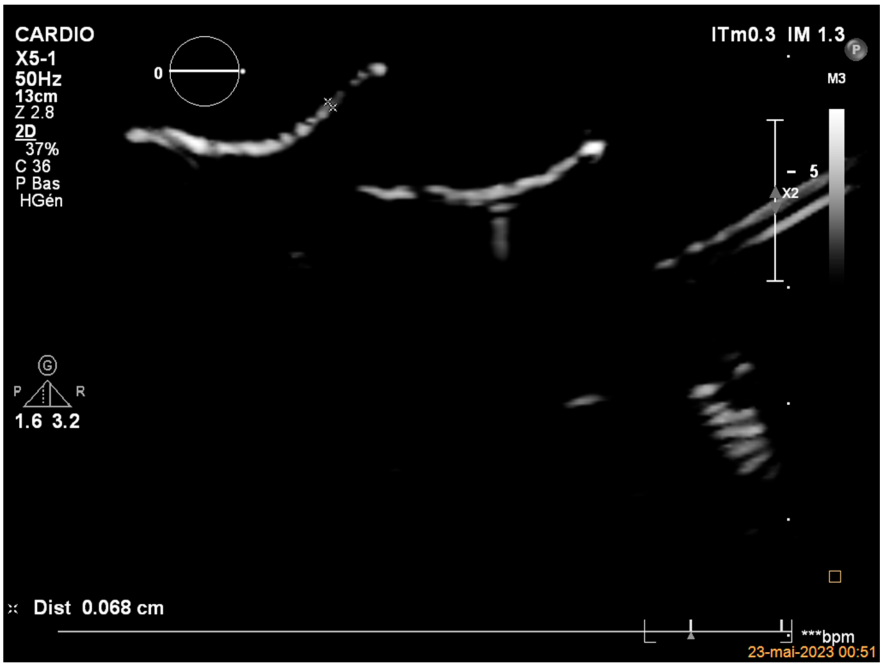

Figure S6: Ultrasound 3D Rendering of Aortic Valve: A Comparison with STL Model

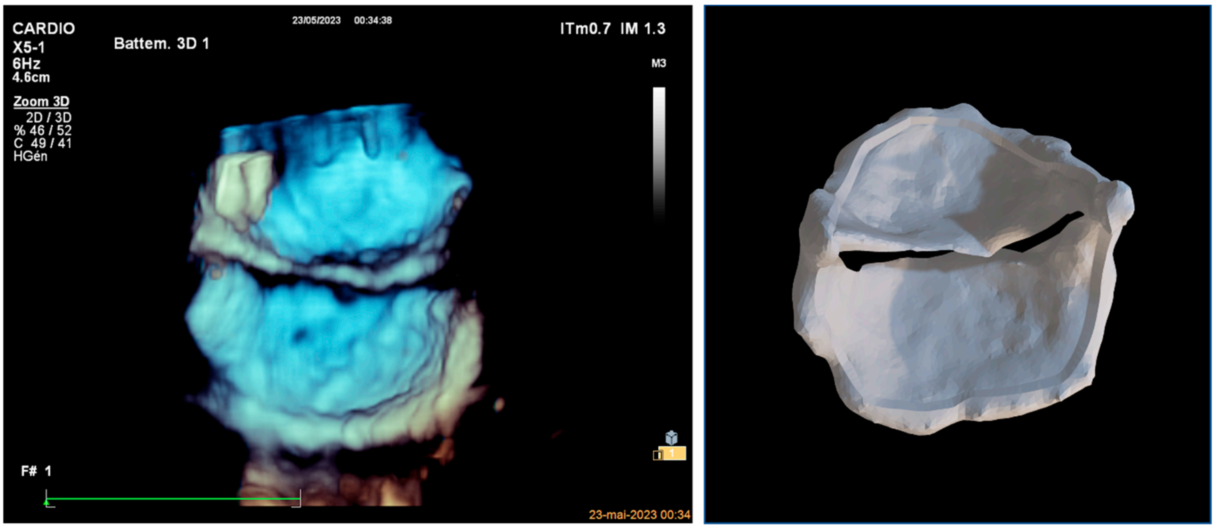

Supplement: Supplementary file 1 [file polymers-16-00900-s001.zip › polymers-2831228-supplementary.pdf]
